# Seguimiento sintomatológico a largo plazo de pacientes con síndrome de COVID persistente

**DOI:** 10.1016/j.aprim.2025.103425

**Published:** 2026-01-16

**Authors:** Andrés Carrascosa Gil, Miriam Gabella Martín, María del Camino Salazar Lozano, Eduardo Tamayo Gómez, Jesús Rico Feijoo, César Aldecoa Álvarez de Santullano

**Affiliations:** a Anestesia y Reanimación, Hospital Universitario Río Hortega, Valladolid, España; b Medicina Interna, Hospital Universitario Río Hortega, Valladolid, España; c Medicina Familiar y Comunitaria, Centro de Salud de Carbonero el Mayor, Segovia, España; d Anestesia y Reanimación, Hospital Clínico Universitario, Valladolid, España

**Keywords:** Síndrome de COVID persistente, Seguimiento a largo plazo, Síntomas, Escalas, Long COVID Syndrome, Long-term follow-up, Symptoms, Scales

## Abstract

**Objetivo:**

El objetivo principal del estudio fue investigar acerca de la evolución sintomatológica a largo plazo del COVID persistente. El objetivo secundario del estudio fue valorar si algún tratamiento tuvo impacto en la evolución sintomatológica.

**Diseño:**

Estudio longitudinal, prospectivo, observacional, no controlado.

**Emplazamiento:**

Servicio de Medicina Interna del Hospital Universitario Río Hortega, Valladolid, España.

**Participantes:**

Cuarenta pacientes con COVID persistente (casos) y 40 voluntarios (controles) de la misma edad y sexo que los casos, que hubieran pasado COVID agudo sin desarrollar COVID persistente.

**Mediciones principales:**

Se enviaron por correo postal unas escalas, índices y cuestionarios para evaluar los principales síntomas de COVID persistente. Se analizaron fatiga (MFIS), trastornos emocionales (HADS), trastornos del sueño (PSQI), alteraciones cognitivas (MFE-30), disnea (mMRC), ejercicio físico (GPAQ), calidad de vida (SF-36) y dolor (EGDC). Los casos fueron reevaluados a los tres años.

**Resultados:**

A los tres años los casos registraron una mejoría en la disnea (mMRC) que pasó de 1,38 a 1,10 (p = 0,014). Hubo 10 casos que refirieron haber seguido como tratamiento ejercicio físico aeróbico y/o anaeróbico y se obtuvo que en el dolor (EGDC) su grado de severidad del dolor mejoró pasando de 2,7 a 1,3 (p = 0,039). En el resto de las mediciones las variaciones en los casos no alcanzaron la significación estadística.

**Conclusiones:**

Tras tres años de seguimiento los pacientes con COVID persistente continúan con puntuaciones sintomatológicas similares a las iniciales y peores que las del grupo control. El tratamiento con ejercicio físico aeróbico y/o anaeróbico puede contribuir a mejorar parcialmente su evolución sintomatológica.

## Abstract (en)

**Objective:**

The primary aim of the study was to investigate the long-term symptom progression of long COVID. The secondary objective was to assess whether any treatment had an impact on symptom evolution.

**Design:**

Longitudinal, prospective, observational, uncontrolled study.

**Setting:**

Internal Medicine Department, Río Hortega University Hospital (Valladolid).

**Participants:**

40 patients with long COVID (cases) and 40 volunteers (controls) of the same age and sex as the cases, who had experienced acute COVID-19 without developing long COVID.

**Main measurements:**

Scales, indices, and questionnaires were sent by post to evaluate the main symptoms of long COVID. The following were assessed: fatigue (MFIS), emotional disorders (HADS), sleep disturbances (PSQI), cognitive impairments (MFE-30), dyspnea (mMRC), physical exercise (GPAQ), quality of life (SF-36), and pain (CPGS). The cases were reassessed after three years.

**Results:**

After three years, cases showed an improvement in dyspnea (mMRC), which decreased from 1.38 to 1.10 (p = 0.014). Ten cases reported having followed aerobic and/or anaerobic physical exercise as treatment, and it was observed that their pain severity (CPGS) improved from 2.7 to 1.3 (p = 0.039). In the remaining measurements, variations among cases did not reach statistical significance.

**Conclusions:**

After three years of follow-up, patients with long COVID continued to have similar symptom scores to their initial assessments, and worse than those of the control group. Aerobic and/or anaerobic physical exercise may contribute to partially improving symptom evolution.

## Introducción

Se calcula que tras la fase aguda del COVID-19 aproximadamente un 10% de los pacientes no se recuperan completamente persistiendo algunos síntomas en el tiempo pasados tres meses. A esta entidad nosológica denominada Long COVID / COVID persistente^1^ se le ha ido prestando progresivamente una mayor atención. El COVID persistente es un síndrome complejo que afecta a múltiples órganos y sistemas^1^, ^2^, ^3^, ^4^. Según la definición de consenso^5^ alcanzada por la Organización Mundial de la Salud (OMS) en 2021 el COVID persistente se define como un cuadro clínico «con antecedentes de infección probable o confirmada por SARS-CoV-2, generalmente tres meses después del inicio de la COVID-19, con síntomas que persisten durante al menos dos meses y no pueden explicarse por un diagnóstico alternativo».

Se han descrito hasta 200 síntomas diferentes^4^ de COVID persistente. En el presente estudio se seleccionaron y analizaron a través de índices, escalas y cuestionarios, los ocho principales según la *Guía Clínica para la Atención al Paciente Long COVID / COVID Persistente*^6^. Estos síntomas son: fatiga, trastornos emocionales, trastornos del sueño, trastornos de atención y de memoria, disnea, disminución de la actividad física, deterioro de la calidad de vida y dolor crónico. Los índices, escalas y cuestionarios de medición en salud son herramientas que permiten medir algunas características de los pacientes que son difíciles de medir objetivamente^7^.

El objetivo principal del estudio fue investigar acerca de la evolución sintomatológica a largo plazo del COVID persistente. La pregunta principal que responder fue: ¿a los tres años se aproximan los principales síntomas de los pacientes con COVID persistente a los de los pacientes que pasaron la infección aguda por COVID sin llegar a desarrollar COVID persistente?

El objetivo secundario del estudio fue valorar si algún tratamiento seguido por los pacientes con COVID persistente tuvo impacto en la evolución sintomatológica. La pregunta en este caso fue: ¿hay diferencias en la evolución sintomatológica entre los pacientes con COVID persistente que han seguido un tratamiento y los que no?

## Material y métodos

Estudio observacional longitudinal de casos y controles. Como criterios de inclusión en el estudio se seleccionaron a pacientes mayores de 18 años y menores de 75 años que aceptasen la inclusión en el estudio y que hubiesen padecido infección aguda por COVID-19. Los casos debían haber recibido el diagnóstico de COVID persistente y fueron reclutados de manera no aleatorizada de la lista de pacientes atendidos por COVID persistente en el Servicio de Medicina Interna del Hospital Universitario Río Hortega, Valladolid. Se realizó un emparejamiento artificial de los casos con controles. Se seleccionaron como controles a personas voluntarias de la misma edad (con un intervalo de -2 años a + 2 años) y del mismo sexo que cada caso. Los controles debían haber pasado la infección aguda por SARS-CoV-2 sin desarrollar COVID persistente. Para su selección se preguntó a los casos por familiares o conocidos de su entorno con las características mencionadas. Los criterios de exclusión del estudio fueron la negativa a participar en el estudio en cualquier momento de este y, en los casos, la revocación del diagnóstico de COVID persistente durante la realización del estudio. Un paciente inicialmente incluido como caso fue excluido del mismo por la aparición de un cáncer colorrectal lo que supuso la revocación de su diagnóstico inicial de COVID persistente.

El reclutamiento fue de marzo a mayo de 2022. Para determinar el tamaño muestral del estudio se realizó primero un estudio piloto con 10 pacientes casos con COVID persistente y 10 pacientes del grupo control. Para un error de tipo 1 de 0,05 y una potencia de 80% se calculó un tamaño muestral de n = 33. Se estimó un 20% de pérdidas en el seguimiento y se redondeó al alza n = 40 (en cada grupo). En la segunda evaluación de los casos, a los tres años, no hubo ninguna pérdida en el seguimiento ni abandono.

En la evaluación a los tres años de los casos se descartaron aquellos tratamientos que tuvieran una n ≤ 5 por carecer el estudio comparativo correspondiente de potencia estadística suficiente para el análisis estadístico.

Para la evaluación de la fatiga se utilizó la escala de modificada del impacto de la calidad de vida (MFIS)^8^. Respecto la evaluación de los trastornos emocionales se empleó la escala de ansiedad y depresión hospitalaria (HAD)^9^. En cuanto a los trastornos del sueño para su evaluación se empleó el índice índice de calidad del sueño de Pittsburgh (PSQI)^10^. Para la medición de los trastornos de atención y de memoria se utilizó el cuestionario modificación del cuestionario de fallos de memoria en la vida cotidiana (MFE-30)^11^. Para la medición de la disnea se empleó la escala modificada de la disnea (mMRC)^12^. El cuestionario global sobre actividad física (GPAQ)^13^ fue utilizado para evaluar los niveles de actividad física. Para evaluar el posible deterioro de la calidad de vida se utilizó el cuestionario de salud de formato breve (SF-36)^14^. Por último, se analizó el dolor crónico a través de la escala española de gradación de dolor crónico (EGDC)^15^.

Las variables cualitativas se presentan como proporciones y las cuantitativas se presentan como medias (desviación estándar). Las comparaciones entre grupos de variables continuas fueron realizadas a través de la prueba *t* de Student. Las comparaciones para las variables categóricas fueron realizadas a través del test de *X*^*2*^ de Pearson (o la prueba exacta de Fisher si no se cumplían las condiciones para la aplicación del test *X*^*2*^ de Pearson). Antes de la realización de las pruebas paramétricos, la homogeneidad de varianzas fue analizada utilizando el test de Levene. Si las varianzas no eran homogéneas se aplicó la prueba de Welch. Para evaluar la normalidad se realizó la prueba de Saphiro Wilk. Todos los valores p se calcularon a dos colas y el nivel de significación estadística se estableció en 0,05. Para el análisis estadístico se utilizó el programa SPSS® versión 15.0.

Se obtuvo el consentimiento informado de todos los participantes y el proyecto de investigación fue aprobado por el Comité de Ética de la Investigación con medicamentos CEIm 21-PI197.

El estudio fue registrado en clinicaltrials.gov con el número de referencia NCT06879535.

**Figure:**
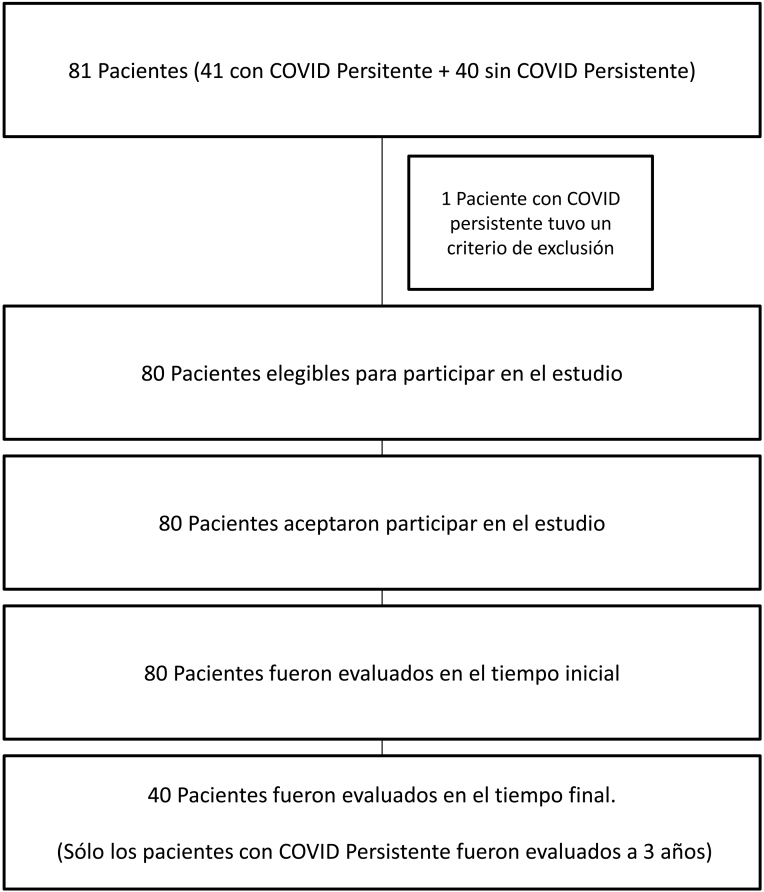
**Esquema general del estudio**.

## Resultados

**Figura 1:**
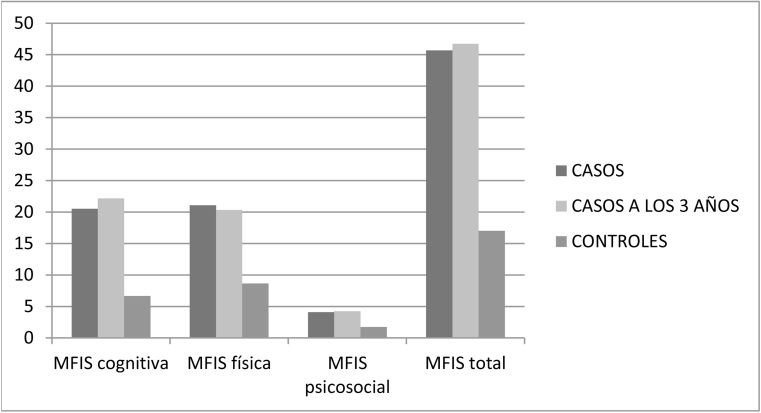
Muestra los resultados de los valores de MFIS cognitiva, MFIS física, MFIS psicosocial y MFIS total en los casos en el momento del diagnóstico, los casos a los 3 años y los controles. MFIS: escala modificada del impacto de la calidad de vida.

Para evaluar el objetivo principal, se realizó un análisis estadístico comparando diversas variables del grupo de los casos y del de los controles (tabla 1). Respecto de la fatiga, en la escala MFIS (fig. 1) en los casos se aprecia que tras tres años la MFIS cognitiva pasó de 20,53 a 22,18 (p = 0,070), MFIS física de 21,08 a 20,35 (p = 0,400), MFIS psicosocial de 4,08 a 4,23 (p = 0,613) y MFIS total de 45,68 a 46,75 (p = 0,541). Los controles obtuvieron resultados inferiores a los casos en MFIS cognitiva 6,65 (p = 0,000), MFIS física 8,65 (p = 0,000), MFIS psicosocial 1,72 (p = 0,001) y MFIS total 17,02 (p = 0,000).

**Tabla 1:** Muestra la comparación de diversas variables del grupo de los casos y del de los controles

	Casos (n = 40) Media (DE)	Controles (n = 40) Media (DE)
*Edad (años)*	52,93 (13.196)	53,18 (12.997)
*Diferencia (Valor p)*		*0,250 (0,263)*
*Sexo*		
Varón: n (%)	19 (47,5%)	19 (47,5%)
Mujer: n (%)	21 (52,5%)	21 (52,5%)
*X*^*2*^*de Pearson (Valor p)*		*0,000 (1.000)*
Peso (kg)	73.775 (14.687)	72.575 (12.895)
*Diferencia (Valor p)*		*-1.200 (0,524)*
Altura (cm)	167.650 (9.161)	170.225 (9.864)
*Diferencia (Valor p)*		*2.575 (0,083)*
IMC	26.141 (4.191)	24.990 (3.589)
*Diferencia (Valor p)*		*-1.150 (0,111)*
*Fumador*		
No fumador: n (%)	27 (67,5%)	27 (67,5%)
Exfumador: n (%)	10 (25%)	8 (20%)
Activo: n (%)	3 (7,5%)	5 (12,5%)
*X*^*2*^*de Pearson (Valor p)*		*0,722 (0,697)*
*Consumo de alcohol*		
No: n (%)	20 (50%)	22 (55%)
Sí: n (%)	20 (50%)	18 (45%)
*X*^*2*^*de Pearson (Valor p)*		*0,201 (0,654)*
*Tratamiento con antidepresivos y/o ansiolíticos*		
No: n (%)	35 (87,5%)	38 (95%)
Sí: n (%)	5 (12,5%)	2 (5%)
*(Valor p del estadístico exacto de Fisher)*		*(0,432)*
*Asma*		
No: n (%)	34 (85%)	36 (90%)
Sí: n (%)	6 (15%)	4 (10%)
*X*^*2*^*de Pearson (Valor p)*		*0,457 (0,499)*
*Diabetes Mellitus*		
No: n (%)	39 (97,5%)	39 (97,5%)
Sí: n (%)	1 (2,5%)	1 (2,5%)
*(Valor p del estadístico exacto de Fisher)*		*(1.000)*
*Dislipemia y/o hipercolesterolemia*		
No: n (%)	36 (90%)	36 (90%)
Sí: n (%)	4 (10%)	4 (10%)
*(Valor p del estadístico exacto de Fisher)*		*(1.000)*
*Fibrilación auricular*		
No: n (%)	38 (95%)	40 100%)
Sí: n (%)	2 (5%)	0 (0%)
*(Valor p del estadístico exacto de Fisher)*		*(0,494)*
*Hipertensión arterial (HTA)*		
No: n (%)	30 (75%)	34 (85%)
Sí: n (%)	10 (25%)	6 (15%)
*X*^*2*^*de Pearson (Valor p)*		*1.250 (0,264)*

DE: desviación estándar; IMC: índice de masa corporal; HTA: hipertensión arterial.

Los casos y los controles no presentaban diferencias estadísticamente significativas en las variables descritas.

En lo que respecta a los trastornos emocionales, en la escala de HAD, en los casos se obtuvo que tras tres años HAD ansiedad pasó de 8,90 a 8,60 (p = 0,500) y HAD depresión de 7,58 a 6,95 (p = 0,168). Los controles obtuvieron unos valores inferiores a los de los casos en HAD ansiedad 3,65 (p = 0,000) y HAD depresión 2,27 (p = 0,000).

En cuanto a los trastornos del sueño, en el índice PSQI, en los casos se obtuvo que tras tres años PSQI global pasó de 10,63 a 9,50 (p = 0,050). En los controles se observaron unos valores inferiores a los de los casos en todos los componentes del PSQI y el PSQI global fue 5,77 (p = 0,000). Respecto de los trastornos de atención y de memoria, en el cuestionario MFE-30 se observó que, en los casos tras tres años, MFE-30 funcionamiento general pasó de 22,75 a 24,48 (p = 0,337), MFE-30 actividades de la vida diaria de 14,68 a 14,75 (p = 0,907), MFE-30 total de 37,43 a 39,23 (p = 0,416) y la corrección alternativa de MFE-30 de 6,18 a 6,40 (p = 0,733). Los controles obtuvieron unos valores inferiores a los casos para MFE-30 funcionamiento general (6,65; p = 0,000), MFE-30 actividades de la vida diaria (5,87; p = 0,000), MFE-30 total (12,52; p = 0,000) y en la corrección alternativa del MFE-30 (0,82; p = 0,000).

En cuanto a la disnea, en la escala mMRC, los casos obtienen en el momento inicial 1,38 y este resultado mejora a los tres años pasando a 1,10 (p = 0,014). El valor obtenido por los controles (0,43) fue sensiblemente inferior a los casos de manera estadísticamente significativa (p = 0,000).

Respecto de la actividad física, en el cuestionario GPAQ, se observó que, en los casos tras tres años, MET-minutos de actividad semanal pasó de 5.792,82 a 6.228,21 (p = 0,609), actividad física total de media diaria de 176,48 a 191,79 (p = 0,545) y tiempo sedentario total de media diario de 296,89 a 293,11 (p = 0,836). Por su parte los controles obtuvieron unos valores inferiores a los de los casos en METS-minutos actividad física semanal (3.258,97; p = 0,006) y actividad física total de media diaria (98,71; p = 0,005). El tiempo sedentario que fue superior en los controles (378,64; p = 0,038).

**Figura 2:**
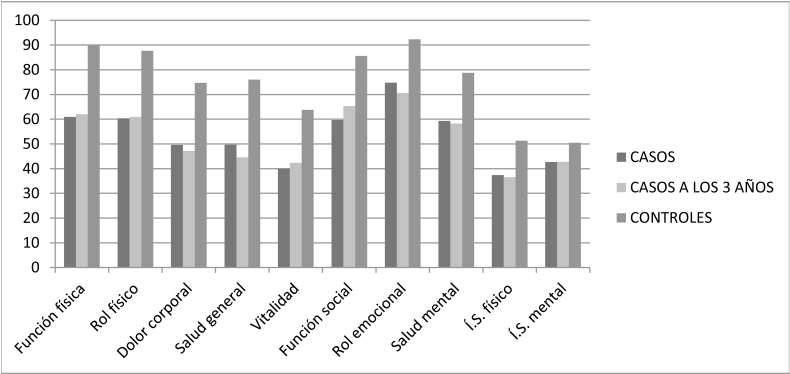
Muestra los resultados del cuestionario SF-36 en los casos en el momento del diagnóstico, los casos a los 3 años y los controles. SF-36: cuestionario de salud de formato breve SF-36.Í.S. físico: índice sumario físico; Í.S. mental: índice sumario mental.

En cuanto a la calidad de vida, en el cuestionario SF-36 (fig. 2) se observó que en los casos tras tres años función física transformada pasó de 60,87 a 62,00 (p = 0,688), rol físico transformado de 60,31 a 60,93 (p = 0,837), dolor corporal transformado de 49,57 a 47,12 (p = 0,507), salud general de 49,65 a 44,55 (p = 0,033), vitalidad transformada de 40,00 a 42,34 (p = 0,494), función social transformada de 59,68 a 65,31 (p = 0,155), rol emocional transformado de 74,79 a 70,62 (p = 0,181), salud mental transformada de 59,25 a 58,25 (p = 0,646), índice sumario físico transformado de 37,32 a 36,53 (p = 0,500), índice sumario mental transformado de 42,62 a 42,72 (p = 0,940) y evolución declarada de 2,45 a 3,23 (p = 0,014). Los controles obtuvieron unos valores superiores a los casos en función física transformada 89,75 (p = 0,000), rol físico transformado 87,65 (p = 0,000), dolor corporal transformado 74,67 (p = 0,000), salud general transformada 76,02 (p = 0,000), vitalidad transformada 63,75 (p = 0,000), función social transformada 85,62 (p = 0,000), rol emocional transformado 92,29 (p = 0,000), salud mental transformada 78,75 (p = 0,000), índice sumario físico transformado 51,32 (p = 0,000), índice sumario mental transformado 50,51 (p = 0,000) y evolución declarada 2,77 (p = 0,140).

En cuanto al dolor crónico, en la escala EGDC en los casos tras tres años valor P1 pasó de 90,54 a 101,49 (p = 0,246), puntuación de 28,94 a 29,48(p = 0,766), intensidad característica del dolor de 44,38 a 47,58 (p = 0,281), puntuación de discapacidad de 35,97 a 36,16 (p = 0,952), clasificación de discapacidad de 2,03 a 2,08 (p = 0,866) y grado de severidad del dolor de 2,10 a 1,74 (p = 0,235). En los controles el valor obtenido en todos los parámetros de la escala EGDC era inferior a los casos: valor P1 (32,54; p = 0,000), puntuación (10,65; p = 0,000), intensidad característica del dolor (22,30; p = 0,000), puntuación de discapacidad (9,91; p = 0,000), clasificación de discapacidad (0,60; p = 0,001), y grado de severidad del dolor (1,12; p = 0,002).

Respecto a los resultados de evaluación del objetivo secundario, únicamente un tratamiento alcanzó una n > 5: el ejercicio físico aeróbico y/o anaeróbico (n = 10). En primer lugar, se realizó un análisis estadístico comparando diversas variables del grupo de los casos que realizaron ejercicio físico y el de los casos que no realizaron ejercicio (tabla 2).

**Tabla 2:** Muestra la comparación de diversas variables del grupo de los casos que realizaron ejercicio físico aeróbico y/o anaeróbico como tratamiento y el grupo de los casos que no realizaron dicho tratamiento

	Casos ejercicio (n = 10) Media (DE)	Casos no ejercicio (n = 30) Media (DE)
Edad (años)	47,10 (17.798)	54,87 (10.966)
*Diferencia (Valor p)*		*7.767 (0,219)*
*Sexo*		
Varón: n (%)	6 (60%)	13 (43,3%)
Mujer: n (%)	4 (40%)	17 (56,6%)
*X*^*2*^*de Pearson (Valor p)*		*0,835 (0,361)*
Peso (kg)	78.300 (13.548)	72.266 (14.957)
*Diferencia (Valor p)*		*-6.033 (0,266)*
Altura (cm)	172.000 (8.082)	166.200 (9.159)
*Diferencia (Valor p)*		*-5.800 (0,083)*
IMC	26.364 (3.577)	26.066 (4.431)
*Diferencia (Valor p)*		*-0,298 (0,848)*

DE: desviación estándar; IMC: índice de masa corporal.

Ambos grupos no presentaban diferencias estadísticamente significativas en las variables descritas.

La única diferencia evolutiva que alcanzó la significación estadística fue el grado de severidad del dolor (EGDC) que tras tres años pasó en los casos que realizaron ejercicio de 2,70 a 1,30 (p = 0,039) mientras que en los casos que no realizaron ejercicio fue 1,90 en ambas mediciones (p = 1.000).

## Discusión

Las escalas, índices y cuestionarios que se han utilizado en el presente estudio son precisamente las preferidas por los pacientes según el estudio realizado por Sanz et al.^16^.

El diagnóstico de COVID persistente es sintomatológico. Al obtenerse el diagnóstico a partir de la clínica referida por el paciente, las diferencias encontradas entre casos y controles en MFIS, HAD, PSQI, mMRC, MFE-30, SF-36 y EGDC eran esperables. Se realizó un emparejamiento artificial de los casos con controles para tener unos valores que sirviesen de referencia y que vendrían a constituir un modelo de los que hubieran obtenido los casos de haberles podido evaluar antes de padecer la infección aguda por COVID. Realizar dicha evaluación a los casos a posteriori probablemente carecería de validez por verse afectado por el sesgo del recuerdo. Teniendo los valores de los controles como referencia se trataba aquí de observar cómo es la evolución a largo plazo de los principales síntomas de los pacientes con COVID persistente, síntoma por síntoma, viendo si mejoraban o no y en qué grado. Tras tres años en los casos en la mayoría de los síntomas han experimentado una discreta mejoría clínica, pero siguen lejos de los valores del grupo control. Estos resultados apuntan en la misma dirección a los encontrados por de Azevedo et al.^17^ aunque el seguimiento de dicho estudio fue de un año. Al igual que en nuestro estudio describen una mejoría en la función pulmonar, pero, a diferencia de nuestros resultados, también mejoraron la fatiga y la calidad de vida. Esto pudo deberse a que contaban con un mayor tamaño muestral (n = 350).

En cuanto al objetivo secundario del estudio, el tratamiento con ejercicio físico aeróbico y/o anaeróbico encontró mayores mejorías sintomáticas, aunque la única que alcanzó la significación estadística fue el grado de severidad del dolor (EGDC). Otros estudios también han encontrado que el ejercicio físico puede producir beneficios en la evolución sintomatológica del síndrome COVID persistente^18^, ^19^, ^20^. Las revisiones sistemáticas y metaanálisis publicados por Zheng et al.^18^ y por Pouliopoulou et al.^19^ sugieren que el ejercicio físico rehabilitador es una estrategia terapéutica eficiente y segura para la mayoría de los síntomas del COVID persistente. El estudio publicado por Humphreys et al.^20^ insiste en la importancia de que la pauta de rehabilitación física sea individualizada y supervisada periódicamente.

Este estudio tiene limitaciones importantes. En cuanto al objetivo principal la distribución de sexo en los casos no coincide con la proporción descrita en el COVID Persistente, por lo que las conclusiones derivadas del mismo deberían extrapolarse con precaución. En el caso concreto de los hallazgos del cuestionario GPAQ una posible explicación es que se haya producido un sesgo del entrevistador. Este cuestionario podría hoy sustituirse por nuevas tecnologías disponibles como dispositivos de registro de actividad física. Estas nuevas tecnologías disponen de múltiples funciones como el contaje del número de pasos diario, la medición de horas de sueño o la estimación del consumo calórico diario en función de la actividad física.

Respecto del objetivo secundario el poder estadístico probablemente sea insuficiente para analizar las diferencias evolutivas alcanzadas con el tratamiento considerado. Son necesarios más estudios con mayor potencia estadística y aleatorizados para tener unos resultados con mayor evidencia científica.

De cara al futuro los programas de rehabilitación con ejercicio físico aeróbico y/o anaeróbico, de manera individualizada y supervisada, se perfilan cada vez más como protagonistas en las estrategias terapéuticas del COVID persistente.

## Conclusiones

Tras tres años de seguimiento los pacientes con COVID persistente han experimentado una discreta mejoría clínica pero sus valores siguen lejos de los obtenidos por el grupo control. El tratamiento con ejercicio físico aeróbico y/o anaeróbico puede contribuir a mejorar parcialmente su evolución sintomatológica.

**Lo conocido sobre el tema**

• El síndrome de COVID persistente es un cuadro clínico con antecedentes de infección probable o confirmada por SARS-CoV-2, tres meses después del inicio, y con síntomas que persisten durante al menos dos meses y no pueden explicarse por un diagnóstico alternativo.
• Es un síndrome complejo que afecta a múltiples órganos y sistemas, con más de 200 síntomas descritos diferentes. Los principales son fatiga, trastornos emocionales, trastornos del sueño, trastornos de atención y de memoria, disnea, disminución de la actividad física, deterioro de la calidad de vida y dolor crónico.

**Lo que aporta este estudio**

• Tras tres años de seguimiento los pacientes con COVID persistente en la mayoría de los síntomas han experimentado una discreta mejoría clínica pero sus valores en las escalas siguen lejos de los obtenidos por el grupo control.
• Respecto a la disnea, los pacientes con COVID persistente a los tres años mejoraron de manera estadísticamente significativa (mMRC) que pasó de 1,38 a 1,10 (p = 0,014).
• Los pacientes que como tratamiento realizaron ejercicio físico aeróbico y/o anaeróbico, en la re-evaluación a los tres años, su grado de severidad del dolor (EGDC) mejoró pasando de 2,7 a 1,3, pudiendo este tratamiento contribuir a mejorar parcialmente la evolución sintomatológica.

## Financiación

La presente investigación no ha recibido ayudas específicas provenientes de agencias del sector público, sector comercial o entidades sin ánimo de lucro.

## Consideraciones éticas

El estudio fue aprobado por el Comité de Ética de la Investigación con medicamentos (CEIm) del Hospital Universitario Río Hortega de Valladolid 21-PI197. Todos los pacientes aceptaron participar y firmaron el consentimiento informado. El estudio fue registrado en Clinical.trial.gov con el número NCT06879535.

## Conflicto de intereses

Los autores declaran ausencia de conflictos de interés.
